# In Vitro Characterization of Reversine-Treated Gingival Fibroblasts and Their Safety Evaluation after In Vivo Transplantation

**DOI:** 10.3390/pharmaceutics16020207

**Published:** 2024-01-31

**Authors:** Carlos Miguel Marto, Mafalda Laranjo, Ana Cristina Gonçalves, Anabela Paula, Joana Jorge, Rui Caetano-Oliveira, Maria Inês Sousa, Bárbara Oliveiros, João Ramalho-Santos, Ana Bela Sarmento-Ribeiro, Manuel Marques-Ferreira, António Cabrita, Maria Filomena Botelho, Eunice Carrilho

**Affiliations:** 1 Institute of Experimental Pathology, Faculty of Medicine, University of Coimbra, 3000-548 Coimbra, Portugal; 2 Institute of Biophysics, Faculty of Medicine, University of Coimbra, 3000-548 Coimbra, Portugal; 3 Institute of Integrated Clinical Practice and Laboratory of Evidence-Based and Precision Dentistry, Faculty of Medicine, University of Coimbra, 3000-075 Coimbra, Portugalecarrilho@fmed.uc.pt (E.C.); 4 Coimbra Institute for Clinical and Biomedical Research (iCBR), Area of Environment, Genetics and Oncobiology (CIMAGO), Faculty of Medicine, University of Coimbra, 3000-548 Coimbra, Portugal; acgoncalves@fmed.uc.pt (A.C.G.); boliveiros@fmed.uc.pt (B.O.); mmferreira@fmed.uc.pt (M.M.-F.); 5 Centre for Innovative Biomedicine and Biotechnology (CIBB), University of Coimbra, 3000-548 Coimbra, Portugal; 6 Clinical Academic Center of Coimbra (CACC), 3004-561 Coimbra, Portugal; 7 Laboratory of Oncobiology and Hematology (LOH) and University Clinic of Hematology, Faculty of Medicine, University of Coimbra, 3000-548 Coimbra, Portugal; 8 Pathology Department, Centro Hospitalar e Universitário de Coimbra, 3000-075 Coimbra, Portugal; 9 Germano de Sousa—Centro de Diagnóstico Histopatológico CEDAP, University of Coimbra, 3000-377 Coimbra, Portugal; 10 Department of Life Sciences, University of Coimbra, 3000-456 Coimbra, Portugal; 11 CNC—Center for Neuroscience and Cell Biology, University of Coimbra, 3000-548 Coimbra, Portugal; 12 Laboratory of Biostatistics and Medical Informatics, Faculty of Medicine, University of Coimbra, 3000-548 Coimbra, Portugal; 13 Institute of Endodontics, Faculty of Medicine, University of Coimbra, 3000-075 Coimbra, Portugal

**Keywords:** cell dedifferentiation, reversine, gingival fibroblasts, stem-like cells, regenerative dentistry

## Abstract

Reversine is a purine derivative that has been investigated with regard to its biological effects, such as its anticancer properties and, mostly, its ability to induce the dedifferentiation of adult cells, increasing their plasticity. The obtained dedifferentiated cells have a high potential for use in regenerative procedures, such as regenerative dentistry (RD). Instead of replacing the lost or damaged oral tissues with synthetic materials, RD uses stem cells combined with matrices and an appropriate microenvironment to achieve tissue regeneration. However, the currently available stem cell sources present limitations, thus restricting the potential of RD. Based on this problem, new sources of stem cells are fundamental. This work aims to characterize mouse gingival fibroblasts (GFs) after dedifferentiation with reversine. Different administration protocols were tested, and the cells obtained were evaluated regarding their cell metabolism, protein and DNA contents, cell cycle changes, morphology, cell death, genotoxicity, and acquisition of stem cell characteristics. Additionally, their teratoma potential was evaluated after in vivo transplantation. Reversine caused toxicity at higher concentrations, with decreased cell metabolic activity and protein content. The cells obtained displayed polyploidy, a cycle arrest in the G2/M phase, and showed an enlarged size. Additionally, apoptosis and genotoxicity were found at higher reversine concentrations. A subpopulation of the GFs possessed stem properties, as supported by the increased expression of CD90, CD105, and TERT, the existence of a CD106+ population, and their trilineage differentiation capacity. The dedifferentiated cells did not induce teratoma formation. The extensive characterization performed shows that significant functional, morphological, and genetic changes occur during the dedifferentiation process. The dedifferentiated cells have some stem-like characteristics, which are of interest for RD.

## 1. Introduction

Reversine [2-(4-morpholinoanilino)-6-cyclohexylaminopurine] (RV) is a purine derivative (2,6-disubstituted purine) that has been demonstrated to be capable of inducing significant biological effects, namely, promoting the dedifferentiation of adult cells and as an anticancer agent [1,2,3]. Ding, Schultz, and collaborators first described the potential of RV in 2004, as it reverted C2C12 myoblasts into multipotent progenitor cells, followed by differentiation in adipocytes and osteocytes [4]. Since then, RV’s dedifferentiation potential has been shown for several cell types, such as fibroblasts, macrophage-like cells, preadipocytes, myoblasts, and osteoblasts [3,5,6]. Additionally, it has been demonstrated that depending on the cell type, RV can also increase the differentiation of BMSCs, ASCs, preadipocytes, macrophage-like cells, and myoblasts [1,2,5]. RV promotes changes in cell morphology, metabolism, cell cycle, cell death, cell proliferation, cell signaling, and changes in gene expression, with an increased expression of reprogramming and pluripotency factors such as Oct4. It downregulates Akt, mTOR, and p70^s6k^ pathways, and promotes changes in cell surface markers and protein expression, RNA export, stress control, ATP production, and enzymatic inhibition, among other cellular effects [1,2,3,7,8,9,10]. Furthermore, RV inhibits the aurora kinases A, B, and C, the mitogen-activated protein kinase kinase 1 (MEK1), the nonmuscle myosin II (NMMII) heavy chain, decreases the expression of pro-inflammatory cytokines, blocks cell proliferation, alters the cell cycle, induces apoptosis and autophagy, and inhibits cell motility and adhesion, justifying the interest in its application as an anticancer agent [10,11,12,13,14].

The dedifferentiated cells obtained after treating adult cells with RV present with increased plasticity, and have a high potential for use in regenerative procedures, such as regenerative dentistry (RD). Tooth decay, tooth loss, periodontal diseases, and dental trauma have an incidence of nearly 100% worldwide, and adversely affect patients’ physical and psychological well-being, in addition to their massive economic costs [15,16,17,18]. However, conventional dentistry could only provide non-biological treatments, with a partial or total replacement of the living tissues with synthetic materials. This paradigm changed with the emergence of RD, which allows for the regeneration of lost or damaged tissues [19]. Although the complete replacement of organs or large defects still represents a major challenge, the partial regeneration of some tissues, such as bone or mucosa, is feasible. In such cases, regeneration is achieved by stimulating different cell types, such as progenitor cells, in combination or not with the use of biomaterials [20,21]. 

Advanced RD protocols are based on two main approaches: (1) bioengineering through the collection, selection, and differentiation of stem cells, and (2) the reproduction of dental embryogenesis [20,22]. The first has been the most explored, and has focused on producing matrices with the proper characteristics and selecting and differentiating the stem cells into the desired target tissues [23,24,25,26]. Among the most studied stem cells, dental-pulp mesenchymal stem cells (DPSCs) can be differentiated into odontoblasts, osteoblasts, and cementoblasts, as well as other specialized cell types [26,27]. Although this approach has been relatively successful, one of its main limitations is obtaining the dental stem cells. The collection of these involves tooth extraction, where only a small amount of stem cells can be obtained, and some cell sub-types exist only in developing teeth. Additionally, a specific cultivation process allowing for their identification and separation is required [3,21,26,28]. This situation led researchers to develop regeneration techniques based on more accessible stem cells, such as bone marrow (BMSCs) and adipose tissue (ASCs). However, to obtain both BMSCs and ASCs, invasive procedures are needed, the number of obtained cells can be low, and there are associated rejection and ethical risks when used for allogenic transplantation [26,29]. Most recently, induced pluripotent stem cells (iPSCs) have been developed through the forced expression of reprogramming factors, and have the potential for use in RD [30,31]. Nevertheless, the teratogenic potential of such cells represents a major concern regarding their clinical use [32]. 

Based on this problem, new sources of stem cells are needed, as they are fundamental for developing the RD field and avoiding the constraints of current methodologies [33,34,35]. Thus, dedifferentiated cells have emerged as an alternative source and have enormous potential, although they have been little explored. This process occurs naturally, for instance, during amphibians’ development and spontaneous dedifferentiation in tumors [36], or it can be induced by several methods, such as cell culture conditions, nuclear transfer, cell fusion, modifications to or induction of gene expression, or chemical inductors with molecules such as RV, myoseverine, prostaglandin E2, or fibroblast growth factor, among others [3,4,36,37,38,39,40,41,42].

Among the cells that have been evaluated as sources for dedifferentiation, fibroblasts, specifically skin fibroblasts [3,9], have been explored. Although they are easily collected, it involves a surgical procedure and the possibility of a scar, so other tissue locations are preferred, such as the oral cavity. Gingival fibroblasts (GFs) are the dominant cell population in gingival tissue. This heterogeneous and versatile cell population is responsible for several important functions, such as extracellular matrix production, tissue remodeling, mediating inflammatory processes, modulating the microenvironment, and their healing capacity. Additionally, fibroblasts share phenotypic and regenerative properties with stem cells, particularly the oral cavity populations. Due to their plasticity, GFs have been used to successfully generate iPSCs and in transdifferentiation processes to obtain osteogenic cells [43,44,45,46]. Also, these cells present with other advantages: easy harvesting at the dental office with rapid regeneration, no ethical or compatibility problems when used as an autologous cell source, and, if we intend to regenerate oral tissues, the same microenvironment [47,48]. Taken together, these characteristics support GFs as ideal target cells for use in obtaining stem-like cells.

To our knowledge, the potential of GFs as a cell source for dedifferentiation has not been explored, so the main aim of this work is to characterize mouse gingival fibroblasts after dedifferentiation with RV. Additionally, we aim to determine whether the dedifferentiated cells acquired stem characteristics, which are of interest for RD procedures.

## 2. Materials and Methods

### 2.1. Primary Culture of Gingival Fibroblasts

Tissue collection was performed on 8-week-old male BALB/c mice immediately after euthanasia, following a previously published protocol [49]. Briefly, the gingival tissue surrounding the incisive teeth was collected, fragmented into 1 mm^3^ pieces, and digested with type I collagenase (Affymetrix, Santa Clara, CA, USA), followed by 0.25% trypsin-EDTA (Sigma, St. Louis, MO, USA). The fragments were placed in a culture dish and covered with heat-inactivated fetal bovine serum (FBS) (Sigma, St. Louis, MO, USA). The next day, Dulbecco’s Modified Eagle Medium (DMEM) (Sigma, St. Louis, MO, USA), supplemented with 20% FBS (Sigma, St. Louis, MO, USA), 1% antibiotic/antimycotic (100 U/mL penicillin and 10 μg/mL streptomycin, Sigma, St. Louis, MO, USA), and 25 μΜ of sodium pyruvate (Gibco, Waltham, MA, USA) was added. When cells reached confluence, the cellular suspension was detached and passed through a 100 μm filter to remove potential tissue fragments. The cells were cultured in standard adherent conditions in a HeraCell 150 incubator (BridgePath Scientific, Frederik, MD, USA) and used for experiments between passages 3 and 20. The cells used for experiments were derived from the same primary culture, and for each experiment, RV-treated cells and controls were used in the same passage. 

### 2.2. Cell Doubling Time

The cell doubling time (DT) was determined by seeding 1 × 10^6^ cells in T25 flasks. After 120 h, the number of cells was determined using the Trypan Blue exclusion method [50]. The cell DT was determined by using the formula: DT = 120 × ln(2)/ln(final cell number/initial cell number). Analysis was performed in duplicate and repeated in at least three independent experiments. 

### 2.3. Reversine Administration

Following the manufacturer’s instructions, RV (Sigma-Aldrich, St. Louis, MO, USA) was solubilized in sterile dimethyl-sulfoxide (DMSO) (Carlo Erba Reagents, Val de Reuil, France) to obtain a solution with a final concentration of 1 μM. Cells were seeded at a density of 4 × 10^4^ cells/cm^2^ and left overnight to allow for cell adhesion. On the next day, RV was administered to the cell culture medium at a concentration of 1 μΜ, 2.5 μΜ, or 5 μΜ, depending on the study group. For the groups without a medium change, RV was administered one or four times (every 24 h), without changing the cell culture medium. In the groups with a medium change, the medium was changed every 24 h, and a new administration of RV was then performed. DMSO administration in the same described conditions was used as the control group. The dedifferentiation protocol had a duration of 96 h. The same dedifferentiation conditions were used for all the experiments. The detailed conditions are presented in Table S1. 

### 2.4. MTT Assay

To evaluate the effect of RV treatment on cell metabolic activity, a 3-(4,5-dimethylthiazol-2-yl)-2,5-diphenyltetrazolium bromide (MTT) assay was performed [51]. Briefly, cell cultures were incubated with a solution of MTT (Sigma M5655, St. Louis, MO, USA) at 0.5 mg/mL, pH 7.4. Formazan crystals were solubilized with 0.04 M of hydrochloric acid (100317, Merck Millipore, Burlington, MA, USA) in isopropanol (Sigma 278475, St. Louis, MO, USA). The absorbance was measured at 570 nm, with a reference filter of 620 nm (Synergy HT, Biotek^®^, Santa Clara, CA, USA). The metabolic activity was determined as the percentage of the RV-treated cells’ metabolic activity correlated with the DMSO-treated cells (control group), considered as 100%. Analysis was performed in duplicate and repeated in at least three independent experiments.

### 2.5. SRB Assay

The protein content was determined using a sulforhodamine B (SRB) assay, as previously described [52]. Briefly, cells were fixed with 1% acetic acid (Sigma 109088, St. Louis, MO, USA) in methanol (Sigma 322415, St. Louis, MO, USA) at 4 °C and dyed with a solution of SRB at 0.5% (Sigma S9012, St. Louis, MO, USA). Excess dye was removed by washing. The protein-bound dye was extracted with a solution of Trizma base at 10 mM, pH 10 (T1503, Sigma, St. Louis, MO, USA). Absorbance was read at 540 nm, with a reference filter of 690 nm. The protein content was determined as the percentage of the RV-treated cells normalized to the DMSO-treated cells’ protein content, considered as 100%. Analysis was performed in duplicate and repeated in at least three independent experiments.

### 2.6. Crystal Violet Assay

The cell’s DNA content was determined using a crystal violet assay [53,54]. The cells were fixed with methanol (Sigma 322415, St. Louis, MO, USA) for 30 min at room temperature. The fixative solution was later removed, and a 0.5% crystal violet solution (Sigma C3886, St. Louis, MO, USA) was added to the plates for 10 min. The excess dye was removed, hydrochloric acid in isopropanol was added to solubilize the dye, and the absorbance was measured at 590 nm with a reference filter of 570 nm. The percentage of the RV-treated cells correlated with the DMSO-treated cells (considered as 100%) determined the DNA content. Analysis was performed in duplicate and repeated in at least three independent experiments.

### 2.7. Cell Cycle

The cell cycle was evaluated using flow cytometry, using labeling with propidium iodide (PI) and RNase for RNA removal [55]. Cell suspensions were fixed in vortex agitation with 70% ethanol (Sigma 24102, St. Louis, MO, USA) and incubated. Then, the supernatant was discarded, and a PI/RNase solution (KIT ImmunoStep, Salamanca, Spain) was added. The detection was performed with an excitation of 488 nm and emission of 582 nm wavelengths. The results are presented as cell percentages at sub-G0, G0/G1, S, and G2/M phases. Analysis was performed in duplicate and repeated in at least three independent experiments.

### 2.8. Cell Morphology

Cell morphology was evaluated using light optical microscopy using both the crystal violet and May–Grünwald–Giemsa stainings. The cells were grown in glass coverslips. After treatment, crystal violet staining was performed as previously described for the DNA content evaluation. For the May–Grünwald–Giemsa staining, the cells were stained with a May–Grünwald solution (Sigma-Aldrich, St. Louis, MO, USA) for 3 min and then a Giemsa solution (Sigma-Aldrich, St. Louis, MO, USA) for 15 min. After rinsing with distilled water, the coverslips were allowed to dry at room temperature. Representative images were acquired using a Nikon Eclipse 80i microscope equipped with a Nikon Digital Camera DXM 1200F at 100× and 500× magnification. Analysis was performed in duplicate and repeated in at least three independent experiments.

### 2.9. Viability and Types of Cell Death

To evaluate cell viability and types of cell death, cells were labeled with annexin-V/propidium iodide (AV/PI) and assessed through flow cytometry, as previously described [56]. Cell suspensions were incubated with binding buffer and 2.5 μL AV-FITC and 1 μL PI (KIT ImmunoStep, Salamanca, Spain), following the manufacturer’s instructions. The detection was performed using a 488 nm excitation laser line and 530/30 nm band pass filter for AV, and 488 nm excitation laser line and 585/42 nm band pass filter for PI in a six-parameter, four-color FACSCalibur flow cytometer (Becton Dickinson, Franklin Lake, NJ, USA). Quantification was performed using at least 25,000 events in a specific software (Paint-a-Gate 3.02, Becton Dickinson, Franklin Lake, NJ, USA). The results are presented as percentages of live, early-apoptotic, late-apoptotic/necrotic, and necrotic cells. Analysis was performed in duplicate and repeated in at least three independent experiments.

### 2.10. Comet Assay

To access DNA double-strand break formation, a comet assay was performed as previously described [57]. Alkaline single-cell gel electrophoresis was completed at a potential difference of 25 V and a current of 1 A for 15 min. The slides were dyed with 20 μg/mL of ethidium bromide (Bio-Rad, Hercules, CA, USA) for 5 min, and excessive dye was washed off with distilled water. A positive control of cells treated with 20 nM hydrogen peroxide (Sigma-Aldrich, St. Louis, MO, USA) was included in every run. The visualization was performed in an inverted fluorescent microscope (Motic), with an excitation length of 546 nm and emission at 580/10. Images were acquired using Motic Images v2.0 software (Motic^®^, Tri-County Pkwy, TX, USA). OpenComet software v.1.3.1 [58] was used for image analysis, and at least 100 randomly selected comets were analyzed per condition. The tail momentum was determined, corresponding to the product of tail length and percentage of DNA present in the comet tail. Analysis was performed in duplicate and repeated in at least three independent experiments.

### 2.11. Mesenchymal Stem Cell (MSC) Markers

The expression of CD11b, CD45, CD90, CD105, CD106, and telomerase reverse transcriptase was evaluated using flow cytometry. Briefly, the cells were incubated with the antibodies against CD11b (BioLegend, San Diego, CA, USA), CD45 (BioLegend, San Diego, CA, USA), CD90 (Santa Cruz Biotechnology, Dallas, TX, USA), CD105 (Santa Cruz Biotechnology, Dallas, TX, USA), CD106 (Santa Cruz Biotechnology, Dallas, TX, USA) and telomerase (Biorbyt, Cambridge, UK), following the manufacturer’s instructions. The results are presented as the percentage (%) of positive cells or as the mean intensity of fluorescence (MIF). Analysis was performed in duplicate and repeated in at least three independent experiments.

### 2.12. Cloning Efficiency

A clonogenic assay was performed as described in [59]. Dedifferentiated cells and controls were plated at 100 to 2000 cells per well in six-well plates. Gingival fibroblasts-conditioned medium for 96 h was used for cell seeding. After 10 days, the cells were fixed with methanol and stained with crystal violet, as previously described. The plates were photographed, and colonies of more than 20 cells were counted. Analysis was performed in duplicate and repeated in at least three independent experiments. 

### 2.13. DNA Methylation

The cells’ methylation status was evaluated in DNA samples by determining the 5-methylcytosine (5-mC) levels using an ELISA assay kit (5-MethylFlash^TM^ Global DNA Methylation 5-mC ELISA Easy Kit, Epigentek, Farmingdale, NY, USA), according to the manufacturer’s protocol. Briefly, genomic DNA was extracted from cells, as previously described [60]. The DNA obtained was used to quantify 5-mC levels. The results are expressed as a percentage of DNA methylation. Analysis was performed in duplicate and repeated in at least three independent experiments.

### 2.14. Cellular Senescence

The senescence-associated-β-galactosidase (SA-β-gal) was evaluated using a Senescence Detection Kit (Canvax Biotech, S.L., Córdoba, Spain) [61]. A positive control was added with oxidative stress-induced senescence, using 200 μM of H_2_O_2_. Briefly, cells were fixed and stained with the kit staining solution to detect SA-β-gal. Then, photographs were taken, and randomly selected ones were used for qualitative analysis. Analysis was performed in duplicate and repeated in at least three independent experiments.

### 2.15. Alkaline Phosphatase Gene Expression and Enzyme Activity

Measurements of enzymatic activity and gene expression analysis were performed to evaluate both alkaline phosphatase (AP) protein and the genetic expressions. 

Alkaline Phosphatase Live Stain (Molecular Probes^®^, Eugene, OR, USA) was used for the enzymatic activity analysis, according to the manufacturer’s instructions. Cells were stained, and images were captured 30 min later using a fluorescence microscope, Leica DM IRE2, EC-Plan Neofluor, with 10× magnification. 

Gene expression analysis was performed using real-time reverse transcription–polymerase chain reaction (RT-PCR). Total RNA was extracted using NZYol reagent (NZYTech, Lisboa, Portugal) and converted into cDNA with a NZY First-Strand cDNA Synthesis Kit (NZYTech, NZYTech, Lisboa, Portugal). The cDNA amplification and quantification was performed with the following primers: *Gapdh*: forward 5′-GACAACTTTGGCATCGTGGA-3′ and reverse 5′-ATGCAGGGATGATGTTCTGG-30; *Alp*: forward 5′-CGCCTATCAGCTAATGCACAACA-30 and reverse 5′-ATGAGGTCCAGGCCATCCAG-3′, in a QuantStudio™ 3 System (ThermoFisher Scientific, Waltham, MA, USA) using Xpert Fast SYBR 2x (GRiSP, Porto, Portugal). The results were normalized to the endogenous gene (*Gapdh*), and the formula 2^−ΔΔCt^ was used to calculate the relative expression. The results are presented as *Alp* expression normalized to *Gapdh* expression. Analysis was performed in duplicate and repeated in at least three independent experiments.

### 2.16. Western Blot

The proteins octamer-binding transcription factors 3 and 4 (Oct-3/4) and NANOG homeobox (NANOG) were evaluated using Western blot. mESCs (cell line E14Tg2a) cultured in 2i medium were used as the positive control [62]. The protocol was performed as previously described in [57]. Incubation with the primary antibodies Oct-3/4 (Santa Cruz Biotechnology, Dallas, TX, USA), NANOG (Santa Cruz Biotechnology, Dallas, TX, USA), and actin (Sigma-Aldrich, St. Louis, MO, USA) was performed overnight at 4 °C. After incubation with the anti-mouse antibody (GE Healthcare, Chicago, IL, USA), the blots were stained with fluorescent reagent elemental chlorine-free (ECF) (GE Healthcare, Chicago, IL, USA). Reading was performed using 9000 Typhoon FLA equipment (GE Healthcare, Chicago, IL, USA). Analysis was performed in duplicate and repeated in at least three independent experiments.

### 2.17. Trilineage Differentiation

The osteogenic, adipogenic, and chondrogenic differentiation capacity of the dedifferentiated cells was evaluated by culturing the cells in a specific inductor medium. For the osteogenic differentiation, 5 × 10^3^ cells/cm^2^ were plated. After 24 h, the cell culture medium was replaced with an osteogenic inductor medium (StemPro^®^ Osteogenesis Differentiation Kit, Gibco^®^, Waltham, MA, EUA). The cell culture medium was replaced every 2–3 days. After 7 days, the osteogenic differentiation was confirmed using Alizarin Red S staining [63,64]. Regarding the adipogenic differentiation, 1 × 10^4^ cells/cm^2^ were plated, and the next day the cell culture medium was changed to adipogenic differentiation medium (StemPro^®^ Adipogenesis Differentiation Kit, Gibco^®^, Waltham, MA, USA). The cell culture medium was replaced every 2–3 days, and after 21 days, Oil Red O dye was used to confirm the adipogenic differentiation [65,66]. The chondrogenic differentiation was evaluated by culturing the cells in micromasses. Briefly, cellular suspensions of 1.6 × 10^7^ cells/mL were prepared, and 5 μL micromasses were placed at the center of each well. After 2 h, the chondrogenic inductor medium (StemPro^®^ Chondrogenesis Differentiation Kit, Gibco^®^, Waltham, MA, USA) was carefully added to each well and renewed every 2–3 days. After 21 days, the chondrogenic differentiation was confirmed using Alcian Blue staining [67,68]. 

### 2.18. Teratoma Formation

For the teratoma formation assay, 8-week-old nude mice (BALB/C nu/nu) were used [69]. Dedifferentiated cells were used after the 5 μΜ 4× W protocol, mESCs cells were used as the positive control, and GFs were used as the negative control. A total of 1 × 10^6^ cells were mixed with 100 μL of Matrigel^®^ Matrix (Corning, Glendale, AZ, USA) and subcutaneously injected into the back of 8-week-old nude mice. Three mice were used per group. Teratomas or the areas of cell injection were resected after 8 weeks, or earlier if the tumor volume reached a mean diameter of 1.2 cm [70]. The samples were fixed with 10% neutral-buffered formalin for 24 h and processed for conventional histology. Staining was performed with hematoxylin and eosin (H&E). Photographs were taken at 20×, 40×, 100×, and 200× magnification, and qualitatively analyzed to determine which tissue types were produced. 

### 2.19. Statistical Analysis

Cell cycle and comet assay statistical analyses were performed using IBM SPSS^®^ version 27 (IBM Corp., Armonk, NY, USA). Regarding the cell cycle analysis, data were presented using the median [quartile1, quartile3] and minimum/maximum for each variable within each group. The data normality was evaluated using the Shapiro–Wilk test. Then, the Kruskal–Wallis test was applied to compare each variable among the analyzed groups. Whenever there were statistically significant differences (defined when *p* < 0.05), a Mann–Whitney U test was applied to compare the control group to each one of the other groups, and was adjusted for multiple comparisons using a Bonferroni correction. For the comet assay analysis, data were presented using the median [quartile1, quartile3] and minimum/maximum for each variable within each group. The Shapiro–Wilk test was used to assess the data normality/non-normality. After that, the Kruskal–Wallis test was used to compare each variable among the groups in the analysis. Whenever there were statistically significant differences (defined when *p* < 0.05), a Mann–Whitney U test was applied to compare all pairwise differences and was adjusted for multiple comparisons using a Benjamini–Hochberg correction. Comet assay plots were obtained using Orange, version 3.32.8 [71].

Prism 9 (GraphPad Software, San Diego, CA, USA) was used for analysis of the remaining assay results. The normal distribution of the data was assessed by means of a Shapiro–Wilk test. For variables with normal distribution, Student’s *t*-test was performed when comparing two variables. A one-factor ANOVA with post hoc analysis using Tukey’s test was performed for more than two comparisons. For variables with non-normal distribution, the Mann–Whitney U test was used to compare two variables. The Kruskal–Wallis test with multiple comparisons was performed using the Mann–Whitney U test with Bonferroni correction for more than two comparisons. A *p*-value < 0.05 was considered statistically significant.

## 3. Results

### 3.1. Gingival Fibroblasts Were Successfully Obtained from the Explants

**Figure 1:**
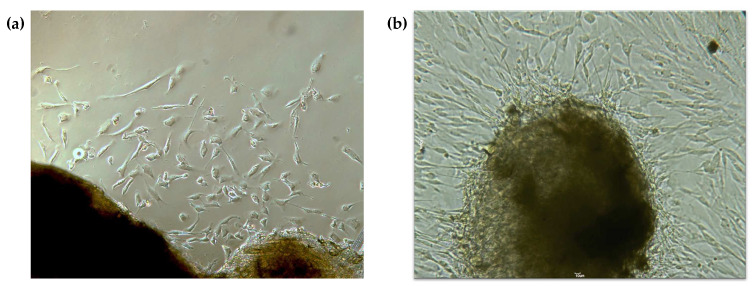
Primary cell culture of gingival fibroblasts. (**a**) Cells migrating from the explant after one week in culture; (**b**) after 15–20 days, an increase in cell number is evident, and cells have acquired a more elongated morphology; 100× magnification.

One week after the cell culture began, cells could be observed migrating from the explants (Figure 1a). These cells presented with typical fusiform morphology (Figure 1b), becoming more elongated with the increase in cell density.

The cell doubling time was 152.95 ± 16.08 h (mean ± SD), determined at passages P3 to P7.

### 3.2. Reversine Induces a Dose-Dependent Response in Metabolic Activity, Protein, and DNA Contents

**Figure 2:**
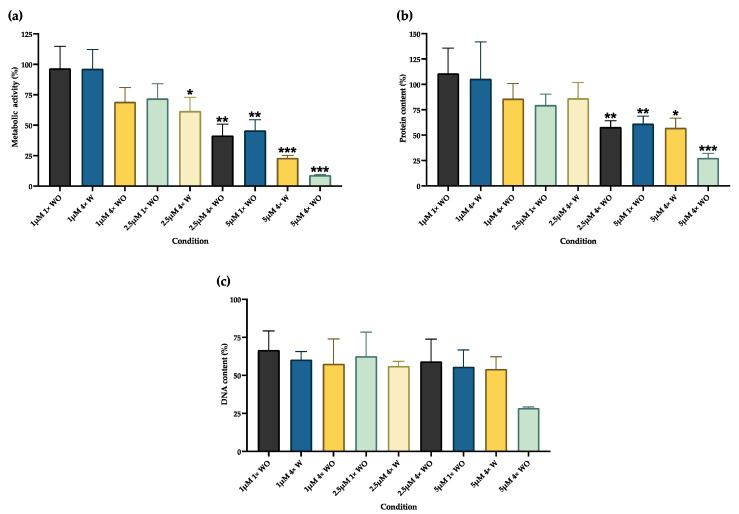
Metabolic activity, protein, and DNA content quantification. (**a**) Metabolic activity was evaluated using an MTT assay; (**b**) protein content using a SRB assay, and (**c**) DNA content using a crystal violet assay. Results are presented as mean ± SE of at least three independent experiments correlated with the control cells’ results. * represents statistically significant differences with the control (DMSO), where * means *p* < 0.05, ** means *p* < 0.01, and *** means *p* < 0.001. 1×: one administration; 4×: four administrations; W: with medium change; WO: without medium change.

RV induces an inhibitory effect on cell function, which is dose dependent, as demonstrated in Figure 2. Regarding the metabolic activity evaluation, the lowest concentrations (1 μM) do not induce any changes in metabolic activity (*p* > 0.05) compared to the DMSO (control group), as seen in Figure 2a. However, for the 2.5 μΜ administered four times (4×) with a medium change (W), an inhibitory effect on metabolic activity occurs (*p* < 0.05). The metabolic activity reduction is more evident for the 2.5 μΜ 4× without a medium change (WO) and the 5 μΜ 1× WO conditions (*p* < 0.01), and for those of the 5 μΜ 4× W and WO (*p* < 0.001). At the 2.5 μΜ 4× concentration, the cumulative effect of RV administration is seen, with the group without a medium change showing a greater reduction in metabolic activity compared to the group with a medium change. The same effect seems to occur in the 5 μΜ groups, although the statistical analysis cannot detect such differences. 

The protein content results are shown in Figure 2b. The 1 μΜ 4× with a medium change group shows an increase in protein content, although it is not statistically significant. A significant decrease in the protein content is evident for the conditions with 2.5 μΜ 4× WO and 5 μΜ 1× WO (*p* < 0.01), 5 μΜ 4× W (*p* < 0.05), and 5 μΜ 4× WO (*p* < 0.001).

Figure 2c presents the cells’ DNA content evaluation. The DNA amount is similar for most conditions, except for the 5 μΜ 4× WO group. 

### 3.3. Reversine Induces Changes in Cell Cycle and Cell Morphology

**Figure 3:**
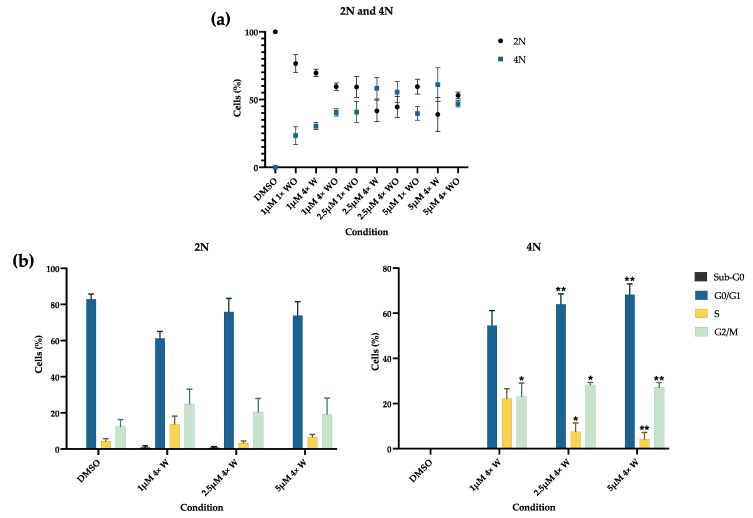
Cell cycle analysis. (**a**) Percentage of cells with 2N and 4N, evaluated using flow cytometry. (**b**) Percentage of cells in Sub-G0, G0/G1, S, and G2/M phases, assessed using flow cytometry. Results are presented as mean ± SE of at least three independent experiments. * represents statistically significant differences with DMSO, where * means *p* < 0.05 and ** means *p* < 0.01. 1×: one administration; 4×: four administrations; W: with medium change; WO: without medium change.

RV leads to the appearance of a population with double the DNA amount, probably due to tetraploid formation, as seen in Figure 3a. For the 2.5 μΜ 4× W condition, the amount of 4N cells is greater than 2N. 

The RV groups were compared to the DMSO for both the 2N and 4N populations. In the 4N population, a significant increase in cells in the G0/G1 phase was seen for the 2.5 μΜ 4× W and 5 μΜ 4× W conditions (*p* < 0.01) (Figure 3b). The cells in the G2/M phase also increased, as was evident for the 1 μΜ 4× W (*p* < 0.05) and the 2.5 μΜ 4× W and 5 μΜ 4× W groups (*p* < 0.01). The increase in the G0/G1 and G2/M phases was accompanied by a significant decrease in the cells in the S phase, both for the 2.5 μΜ 4× W (*p* < 0.05) and the 5 μΜ 4× W (*p* < 0.01) conditions. The same tendency occurred in groups without a medium change, as seen in Figure S1.

**Figure 4:**
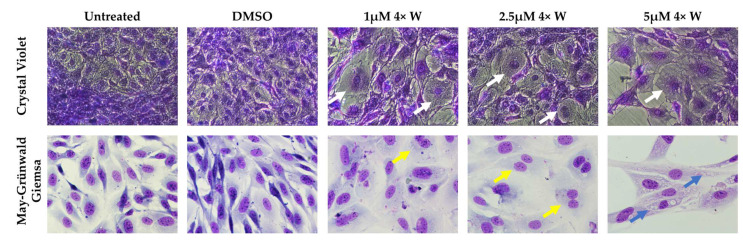
Cell morphology before and after treatment. Crystal violet (**upper row**) and May–Grünwald–Giemsa (**lower row**) stainings are shown. After reversine (RV) administration, an increase in cell size and round shape were evident (white arrows). Binucleated cells were detected (yellow arrows), and at 5 μΜ 4× W, some cells presented with cytoplasmatic vacuolization (blue arrows). 1×: one administration; 4×: four administrations; W: with medium change; Crystal violet: 100× magnification; May–Grünwald–Giemsa: 500× magnification.

The cell cycle changes were accompanied by morphological modifications in some of the cells, and correlated with the administered dose, as seen in Figure 4. The cells’ size increased, and they lost the characteristic elongated morphology of fibroblasts and acquired a round shape. Binucleated cells were detected at an increased number after 2.5 μΜ 4× W administration. Cell death was evident at the higher concentration of 5 μΜ 4× W, as seen by the cellular debris and membrane disruption, while some of the remaining cells presented with cytoplasmatic vacuolization.

### 3.4. Reversine Induces Cell Death by Apoptosis and Genotoxicity at Higher Concentrations

**Figure 5:**
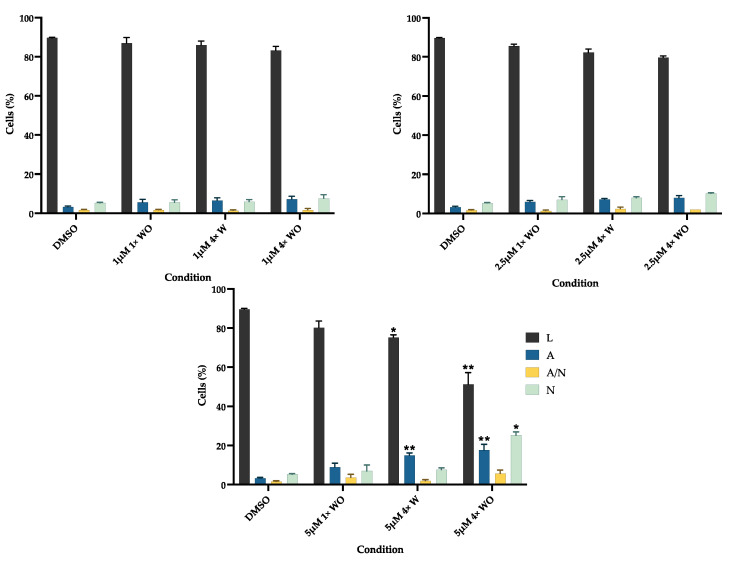
Cell viability and types of cell death. Results were obtained using flow cytometry and are presented as mean ± SE of at least three independent experiments and express the percentage of live cells (L), apoptosis (A), late apoptosis/necrosis (A/N), and necrosis (N). * represents statistically significant differences with DMSO, where * means *p* < 0.05 and ** means *p* < 0.01. 1×: one administration; 4×: four administrations; W: with medium change; WO: without medium change.

To complement the previous results, the viability and cell death types are evaluated and presented in Figure 5. RV promotes cell death through a dose-dependent mechanism, with a higher concentration inducing significant changes. In the 5 μΜ 4× W group, a significant decrease in the live cells (*p* < 0.05) and an increase in the apoptotic cells (*p* < 0.01) can be seen. Regarding the 5 μΜ 4× WO condition, more marked differences occurred, with a significant decrease in the live cells (*p* < 0.01), an increase in the apoptotic cells (*p* < 0.01), and an increase in the necrotic cells (*p* < 0.05). 

**Figure 6:**
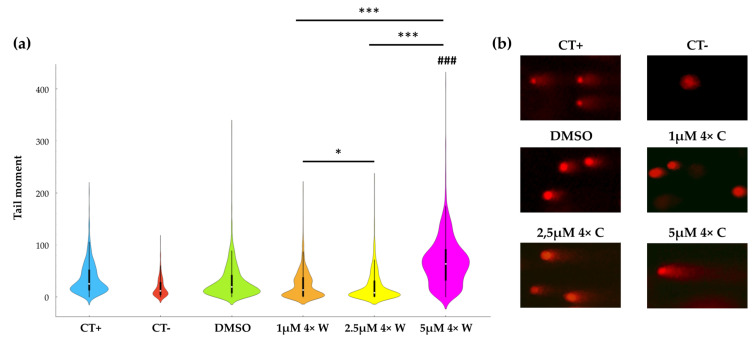
Reversine (RV) genotoxic effect. The RV-induced genotoxicity was evaluated using a comet assay. (**a**) Graphical representation of the tail moment values and distribution. A minimum of 100 comets were evaluated per condition within three independent experiments. (**b**) Representative images of the comet assay results by condition. ^###^ represents statistically significant differences with CT-, where *p* < 0.001 and * represents statistically significant differences between RV groups, where * means *p* < 0.05 and *** means *p* < 0.001. CT+: positive control (H_2_O_2_); CT−: negative control; 1×: one administration; 4×: four administrations; W: with medium change; WO: without medium change; 100× magnification.

In addition to cell death induction, the comet assay results evidence genotoxicity, as seen in Figure 6. There is an increase in DNA damage with increased RV concentrations, as seen by the tail moment. The 5 μΜ 4× W presented with the most significant DNA damage, significantly higher than the 1 μΜ 4× W (*p* < 0.001) and the 2.5 μΜ 4× W (*p* < 0.001) conditions. The higher RV concentration (5 μΜ) also presented with increased genotoxicity with respect to the negative control (*p* < 0.001, indicated with ^###^), while for the 1 μΜ and 2.5 μΜ groups, no statistically significant differences were observed. Interestingly, the tested DMSO concentration (corresponding to a higher RV administration) presented a genotoxic effect per se (*p* < 0.001 with respect to the negative control group), and was even higher than that of the 1 μΜ 4× W and the 2.5 μΜ 4× W groups (*p* < 0.001 for both comparisons). Nevertheless, the 5 μΜ 4× W condition was significantly more genotoxic than that of the DMSO group (*p* < 0.001) and the positive control group (*p* < 0.001). 

### 3.5. Reversine Induced Some Cells to Dedifferentiate and Acquire Stem-like Characteristics

**Figure 7:**
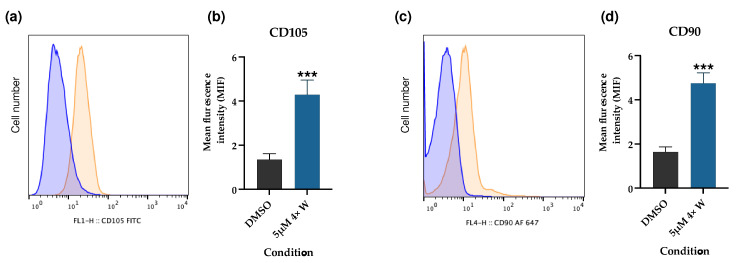
Expression of MSC markers in dedifferentiated cells. (**a**) Representative CD105 expression histogram, evidencing the higher fluorescence intensity of the dedifferentiated cells (orange) with regard to the control (DMSO) (blue); (**b**) mean intensity of fluorescence (MIF) for DMSO and 5 μΜ 4× W groups; (**c**) representative CD90 expression histogram, evidencing the higher fluorescence intensity of the dedifferentiated cells (orange) with regard to the control (DMSO) (blue); (**d**) MIF of CD90 evaluation for both conditions. The results were obtained using flow cytometry and are presented as mean ± SE of at least three independent experiments. *** represents statistically significant differences with DMSO, where *p* < 0.001. 4×: four administrations; W: with medium change.

After the RV treatment, several assays were performed to evaluate the acquisition of stem cell characteristics. Through a flow cytometry analysis, no expression of CD11b or CD45 were detected for either the gingival fibroblasts (controls) or the dedifferentiated cells using the 5 μΜ 4× W protocol. However, regarding CD90 and CD105 expression, both markers showed a significant increase in the 5 μΜ 4× W group (Figure 7). 

**Figure 8:**
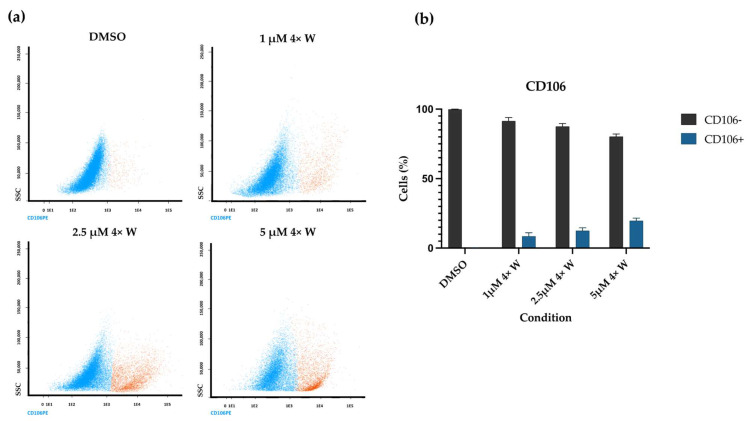
Expression of CD106 on fibroblasts and dedifferentiated cells. (**a**) Representative CD106 dot plots, evidencing the increased number of CD106-positive cells (orange) with regard to the CD 106-negative ones (blue); (**b**) mean cell number of negative and positive CD106 cells for DMSO, 1 μΜ 4× W, 2.5 μΜ 4× W, and 5 μΜ 4× W groups. The results were obtained using flow cytometry and are presented as mean ± SE of at least three independent experiments. 4×: four administrations; W: with medium change.

The CD106 expression is presented in Figure 8. As seen from the dot plots (Figure 8a) and quantification (Figure 8b), the number of CD106-positive cells increases with the increase in RV concentration. For the 5 μΜ 4× W condition, the CD106+ cells correspond to 19.73% of the cell population. 

**Figure 9:**
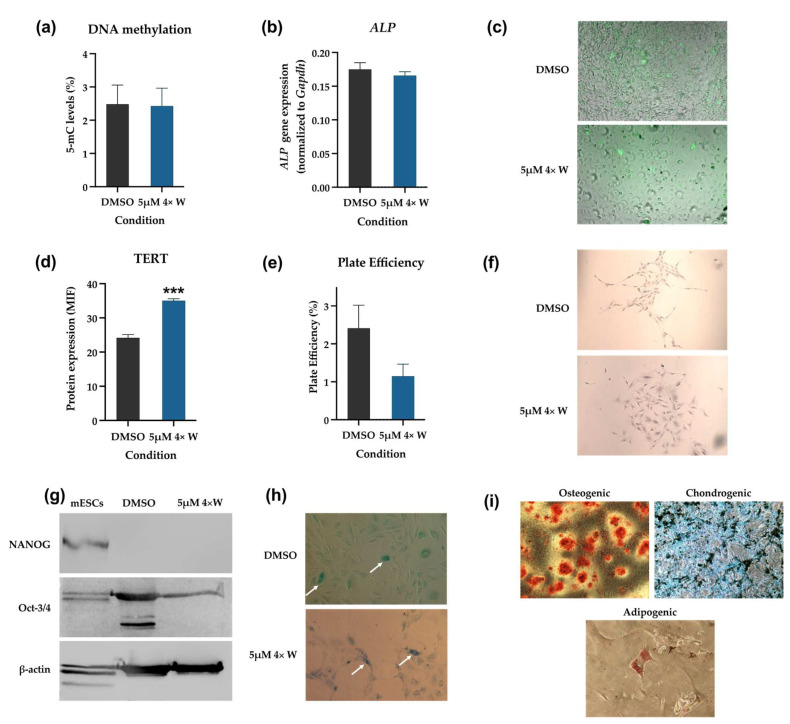
Stem-like cell properties. (**a**) DNA methylation was evaluated using ELISA; (**b**) alkaline phosphatase (ALP) gene expression was assessed using RT-PCR, and (**c**) representative fluorescence microscopy images of enzymatic activity labeling of ALP for each condition, at 100× magnification. (**d**) Telomerase reverse transcriptase (TERT) expression was evaluated using flow cytometry; (**e**) plate efficiency of the two conditions was evaluated using a clonogenic assay, and (**f**) representative light microscopy images of cell colonies, at 40× magnification. (**g**) Analyses of Oct-3/4 and NANOG protein expression were performed using Western blot; (**h**) cell senescence was evaluated through SA-β-gal detection. Senescent cells were identified in both DMSO (control) and 5 μΜ 4× W groups (white arrows), at 100× magnification. (**i**) Representative images of osteogenic differentiation after 7 days, assessed through Alizarin Red S staining (100× magnification); chondrogenic differentiation after 21 days evaluated using Alcian Blue staining (100× magnification); and adipogenic differentiation after 21 days, assessed with Oil Red O staining (400× magnification). For the qualitative evaluation (Figure 8a,b,d,e), the results are presented as mean ± SE of at least three independent experiments. For the plate efficiency determination, six randomly selected wells per experiment were counted. *** represents statistically significant differences with DMSO, where *p* < 0.001. 4×: four administrations; W: with medium change; MIF: mean intensity of fluorescence; mESCs: mouse embryonic stem cells.

Regarding the DNA methylation status, no differences exist between the DMSO group (control) and the 5 μΜ 4× W group, as seen in Figure 9a. Similar results were obtained when evaluating the alkaline phosphatase (ALP) gene expression and enzymatic activity. No statistically significant differences existed between the two groups regarding the *ALP* gene expression (Figure 9b). Also, the qualitative evaluation of enzymatic activity evidenced positive cells in both groups, with similar numbers, as shown in Figure 9c, with some cells displaying high activity. The corresponding channel images are shown in Figure S2.

A different result was seen regarding the telomerase reverse transcriptase (TERT) expression (Figure 9d). A statistically significant increase in TERT expression (*p* < 0.001) was seen after RV 5 μΜ 4× W administration.

Figure 9e presents the results of the plate efficiency evaluation. Colonies of more than 20 cells were counted per condition (representative images are shown in Figure 9f). Although the 5 μΜ 4× W group displayed a decrease in plate efficiency, the difference with regard to the control was not statistically significant.

An evaluation of NANOG and Oct-3/4 using a Western blot evidenced a similar profile for the control and the dedifferentiated cells, as shown in Figure 9g. NANOG expression was not detected in either group, contrary to that of Oct-3/4. Interestingly, the Oct-3/4 expression seems higher in the GFs group (control).

Figure 9h presents a typical image of SA-β-gal detection in the dedifferentiated cells. RV did not induce observable differences in senescence in the treated cells. The positive SA-β-gal cells were a small number in all the analyzed images. 

As presented in Figure 9i, the dedifferentiated cells were able to achieve trilineage differentiation (osteogenic, chondrogenic, and adipogenic). A significant amount of mineralized deposits stained with Alizarin Red S were seen after 7 days of cell culturing in the osteogenic inductor medium. The Alcian Blue staining indicated the deposition of proteoglycans, supporting chondrogenic differentiation. Intracellular lipidic deposits were observed in some cells after staining with Oil Red O, which is an indicator of adipogenic differentiation.

### 3.6. Dedifferentiated Cells Do Not Induce Teratoma Formation

No teratoma formation was observed in the groups of GFs or dedifferentiated cells (5 μΜ 4× W). Animals were euthanized at 8 weeks as planned, and no evidence of viable cells was observed in the injected tissues, confirmed by magnification during the necropsy. However, tumors were observed in the mouse embryonic stem cells (mESCs) group after 1 week. In the same group, at week 4, the tumors achieved critical dimensions, and the animals were euthanized (Figure S3a). After histological processing and analysis, teratoma formation was confirmed by the formation of tissues derived from the three germ layers (Figure S3b–d). 

## 4. Discussion

This is the first report describing the effects of RV in GFs. The extensive characterization performed shows that significant functional, morphological, and genetic changes occurred during the dedifferentiation process. RV presented some toxicity at higher concentrations, with a decrease in metabolic activity and protein content, a cell cycle arrest in the G2/M phase, polyploid formation, morphological changes, cell death by apoptosis, and genotoxicity. Additionally, the dedifferentiated cells increased the expression of CD90, CD105, CD106, and TERT, were able to achieve trilineage differentiation, and did not originate teratomas after in vivo transplantation. These results support the interest in dedifferentiated cells for use in RD. 

A GF culture was successfully established to initiate this study, and a high number of proliferative cells were obtained for the experiments. The existence of a great number of cells is fundamental to RD protocols, and represents the limitation of most stem cell sources. With respect to this, the collection of gingival tissue and the use of GFs represent an advantage. Notably, from a clinical perspective, the collection of gingival tissue is easily performed at the dental office with local anesthesia, the tissue regenerates quickly, and it is a much less invasive procedure than bone marrow or adipose tissue collection, for instance. The gingival tissue also possesses stem cells (gingival mesenchymal stem cells), which can be used in regenerative protocols [72]. However, their number is reduced, and isolation them to obtain a pure stem cell culture requires specific protocols, which are different from the propagation of GFs [73]. 

Since several dedifferentiation protocols using RV are reported, a comprehensive analysis of several administration schemes was initially performed to select the most promising one. RV concentrations from 1 μM to 5 μM were selected and tested with and without a medium change for 4 days, and several assays were performed to evaluate the RV effect on cells. The metabolic activity (MTT assay), protein content (SRB assay), and DNA content (crystal violet assay) results evidenced that RV promoted dose-dependent changes in the cells (Figure 2), indicating a possible toxic effect. This was supported by the differences between groups where the same concentration was tested. When RV was administered four times, the metabolic activity and protein content reduction were higher than when it was administered once. At the same time, when no medium change was performed, the decrease was also higher, probably related to the higher RV concentration in the medium. Similar results with reduced metabolic activity were obtained for other cell types, both for normal and cancer cells [8,74]. However, the reduction depends on the cell type, with some cell types being more sensitive to RV’s effects than others [75]. The DNA content presented few variations between the groups, which can be explained by the changes in cell morphology and polyploidy formation, as further discussed below.

Although the metabolic activity and protein content can indirectly indicate cell viability, RV’s effect on GF viability was further investigated using flow cytometry (Figure 5). The results correlate with the MTT and SRB assays, since cell death was observed at higher concentrations in the groups with and without medium changes. The lower concentration (1 μM) showed no differences in cell viability, as was found in human gingival fibroblast [11]. Similar to the results of the present work, other studies have reported a decrease in cell viability of around 20–30% with 5 μM concentrations, even with minor exposure times [12,74,76,77]. Although necrosis was verified in the 5 μM group without a medium change, apoptosis was the main cell death mechanism, consistent with other findings [12,74,75,77]. Even though caspase expression was not evaluated, previous reports have shown that RV’s effect includes caspase-3 activation and bcl-2 downregulation, thus inducing cell apoptosis both by the intrinsic and the extrinsic pathways [12,74]. Also, aurora kinases are fundamental for cell division regulation. Since RV is an inhibitor of aurora kinases, its effect results in cell cycle arrest and can also lead to cell death [75]. This is also supported by the observed increase in the G0/G1 population (Figure 3b), which suggests the suppression of cell growth through programmed cell death [77]. Taken together, these results suggest that RV acts through a selection process where the remaining viable cells correspond to those that are of interest.

In addition to the abovementioned increase in the G0/G1 population, a decrease in the cell population in the S phase and an increase in the G2/M population were also detected (Figure 3b and Figure S1). This was an expected finding, since most studies on RV’s effects refer to a G2/M arrest [3,5,12,74,75]. At the same time, a 4N population emerged, in which the cell numbers increased with higher RV doses, similar to previous reports [3,7,9]. The G2/M arrest and tetraploid formation mechanism occur due to RV’s effect as an aurora kinase inhibitor. The Aurora kinases family (A, B, and C) regulate fundamental aspects of chromosome segregation and cytokinesis [75,77]. X-ray diffraction crystallography shows that RV binds to the aurora kinase B active site, inhibiting its downstream target histone H3 phosphorylation, and suppressing the protein levels of cyclin D1 (necessary for the cells to enter the next cell cycle from the M phase) and CDK1, while upregulating p21WAF and cyclins B1, cdc2, cdc25c, and D3/CDK6. Altogether, these modifications result in cell cycle arrest, the impairment of cell cytokinesis, and eventual cell death [12,42,75,77,78,79]. In addition to skipping cell division, the cells re-enter the G1 phase and replicate their DNA, thus inducing polyploidy, through a mechanism known as endoreplication [75,78]. Similarly to aurora kinase A, RV was also shown to inhibit aurora kinases B and C, thus contributing to tetraploid formation [79]. Several studies have reported 4N populations above 80%, even superior to the results obtained in the present study [7,9,79]. Again, this indicates that RV’s effects are cell-type dependent, with cancer cells presenting with higher 4N and even 8N populations [77]. This accumulation of cells in G2/M and tetraploid formation may be seen as harmful for the cells since it can later favor cell death; however, these effects on the cell cycle seem to contribute to increased cell plasticity [7]. Polyploidy occurs during the normal development of many organisms, such as mammals, plants, and insects. In normal cells, it may function as an adaptative response to deleterious stimuli, such as genotoxic damage or metabolic stress [7,78]. Since RV was able to promote cell changes in the cell metabolism (Figure 2) and genotoxicity (see Figure 6), it is reasonable to assume the surviving cells entered into an adaptative stage, favoring cell plasticity. At the same time, growth arrest is essential for cell differentiation in many organisms, so RV-induced cell cycle arrest may be fundamental for the cells’ reprogramming process [7,9].

Based on the metabolic activity, protein and DNA contents, cell cycle changes, cell viability, and type of cell death, three conditions (1 μM, 2.5 μM, and 5 μM administered four times with a medium change) were further selected to proceed with the experiments. 

Next, the cell morphology was investigated (Figure 4), which supported the previously discussed results. The cells were enlarged, with a round shape, and some were binucleated and with a multi-lobed nucleus, and peaked at a 5 μM concentration, similar to previous reports [1,6,8,76]. The existence of cells with more than one nucleus justifies the DNA content results (Figure 2c), which remained constant between the different groups, although cell death was observed at higher RV concentrations (Figure 5). The increase in cell size is explained by the previously discussed failure in cytokinesis, and by an active remodeling and organization of the cytoskeleton, with tubulin increase [7]. Interestingly, intracellular vacuoles were seen in the 5 μM 4× group with a medium change (Figure 4), indicating an eventual autophagic process. Lee et al. found similar results in oral squamous cell carcinoma cells [77], and Hu et al. in thyroid cancer cells [13]. The latter confirmed RV-induced autophagy and autophagic influx through the suppression of the Akt/mTOR/p70S6 pathway and the overexpression of LC3-II [13].

In addition, the genotoxicity of RV was also investigated (Figure 6). Besides inducing a higher percentage of cell death and cell cycle changes, RV at 5 μM also induced the highest genotoxicity, determined using the comet assay. These results agree with the previous experiments, since RV’s toxic effect has been observed to be dose-dependent. Also, Hsieh et al. showed that polyploid tumor cells become more sensitive to chemotherapeutic agents and gamma-irradiation due to their higher genetic instability [78]. Since the 5 μM group has a higher percentage of polyploid cells (Figure 3a), it can be assumed that the genotoxic effects of RV and DMSO are more marked in this group. RV was administered using DMSO as the vehicle. DMSO was observed to have a genotoxic effect per se at the higher tested administration volume, corresponding to the volume administered in the 5 μM group (Figure 6). This genotoxicity is even higher than that observed in the 1 μM and 2.5 μM groups, and certainly contributes to the higher genotoxicity seen in the 5 μM group. Although DMSO is well tolerated by the GFs in the lower tested concentrations (similar to other cell types), with irrelevant differences regarding the non-treated cells, DMSO can cause direct toxic effects, in addition to inhibiting the drug-metabolizing enzymes necessary for the metabolic activation or detoxification of chemical agents, thus helping to interpret the results [80,81,82]. Considering these changes in the cell cycle and the genotoxicity of the higher RV concentrations, it will be relevant for future studies to analyze whether changes in the karyotype occur during the dedifferentiation process.

The 5 μM concentration administered four times with a medium change was further selected to continue the analysis. This condition was chosen considering the obtained 4N population and the observed cell death. Next, a set of experiments to characterize the stem properties of the dedifferentiated cells was performed.

The expression of CD105, CD73, and CD90, and the lack of expression of CD45, CD34, CD14 or CD11b, CD79α or CD19, and HLA-DR, together with their adherence to plastic in standard culture conditions, are considered the minimal criteria for defining MSCs [83]. Since the adherence to plastic was already confirmed during the cell culture, the expression of CD105, CD90, CD45, and CD11b was further investigated using flow cytometry. A marked increase in CD90 and CD105 expression were observed (Figure 7), while CD11b and CD45 remained undetectable. This is an important result, since it supports the idea that the dedifferentiated cells present with some stem-like properties compared to the GFs, thus increasing their plasticity. Similar results were obtained for the ear marginal tissue of Luxi cattle fibroblasts with an increased expression of mesenchymal markers CD73, CD44, and CD29 [7]; for annulus fibrous cells with an increased expression of CD90, CD29, and CD44 [8]; and for preadipocytes with an increased mRNA expression of CD73 and CD90 [76]. Interestingly, Qu et al. found an increase in the mesenchymal stem cell marker STRO-1, but no differences regarding CD105 expression, supporting once more the cell specificity of RV’s effects [6].

Next, the expression of the cluster of differentiation 106 (CD106) or vascular cell adhesion molecule-1 (VCAM-1) was investigated, which is a cell adhesion protein, particularly relevant for leucocyte adhesion to the vascular endothelium, in addition to other functions [84]. As mesenchymal stem cells (MSCs) and fibroblasts are phenotypically indistinguishable and express similar surface markers, the CD106 was used to differentiate the cell populations, since it is expressed in MSCs but not in fibroblasts or only residually expressed [85,86,87]. A CD106+ population was found, which increased with increased RV concentrations, supporting the idea that some GFs dedifferentiate into stem-like cells. Given the gingival tissue’s cell heterogeny, RV could have acted through a selection process, supporting gingival stem cell proliferation. However, Chen et al. demonstrated, using a clonal analysis, that C2C12 myoblasts grown from single cells in the presence of RV were not derived from a less-differentiated stem-like population [5]. The isolation of this CD106+ population using magnetic cell sorting will allow for the further exploration of whether it produces improved stem properties. Since surface cell markers’ expression can vary under numerous stimuli, such as cell passages, assessing whether CD106 expression is maintained after the dedifferentiation protocol is highly relevant.

Aiming to investigate the dedifferentiated cells further, additional stem properties were evaluated. DNA methylation plays a critical role in stem cell maintenance and lineage commitment during differentiation, and epigenetic changes, such as hypo-methylation status, are characteristic of dedifferentiated cells, which are different from adult cells [88,89]. No difference between the global DNA methylation status of the dedifferentiated and GFs was found (Figure 9a). However, the methylation of specific proteins, for instance, histones, can occur and is worthy of investigation [7]. Specific methylation patterns can also occur depending on cell type, with fibroblasts experiencing a decrease and MSCs an increase in total methylation with cell passaging and aging [90]. In this way, it will be relevant to evaluate the DNA methylation at different passages for both groups. Also, evaluating the cell methylation patterns could provide evidence of more differences between the GFs and dedifferentiated cells, providing new information on the changes that occur during the dedifferentiation process.

Alkaline phosphatase is a pluripotent stem cell marker, and its expression is downregulated when differentiation occurs [91]. Both the gene and protein expression of ALP were evaluated, and no differences were found. Although ALP is widely accepted as a marker of embryonic stem cells, its expression in other stem cell types is inconsistent or very low [91]. TERT expression, an embryonic stem cell marker highly expressed in stem cells, activated lymphocytes, or other highly proliferative cells, was also evaluated [92]. Notably, TERT expression was significantly increased, supporting the increased proliferation capacity of the dedifferentiated cells. Although TERT can also regulate DNA methylation through different mechanisms, our DNA methylation results showed that increased TERT expression was not associated with DNA methylation changes in the dedifferentiated cells [92]. RV has also been shown to decrease the clonogenic capacity of treated cells, especially in p53-deficient cells [93]. Although a decrease in the clonogenic capacity was found, it was not significant, probably due to the normal p53 function of the dedifferentiated cells. However, if the RV concentration or the exposure time increased, substantial changes in the clonogenic capacity could occur, associated with higher RV toxic effects.

After this, the expression of Oct-3/4 and NANOG, two transcriptional factors that regulate embryonic stem cells’ self-renewal and pluripotency capacity, were evaluated [94]. While NANOG expression was not detected, Oct-3/4 was expressed in the GFs (control cells) and dedifferentiated cells (Figure 9g). Similar results were reported for BMSCs, ASCs, and ear marginal tissue fibroblasts [1,7]. These results suggest that Oct-3/4 is fundamental for cells to acquire multipotency capacity, since Oct-3/4 seems to be the most important factor for pluripotency, while NANOG is not necessary for cell reprogramming [94]. The antibody used for the WB experiments can only detect the Oct4A isoform, which is a limitation. Since Oct4B is associated with normal differentiated cells and Oct4A with reprogramming and iPSCs, it would be relevant to evaluate the two isoforms on GFs and dedifferentiated cells in future experiments [95]. Also, the apparently higher expression of Oct-3/4 in the GFs (DMSO group) may reflect the expression of pseudogenes and not the POU5F1 gene [96]. The senescence qualitative evaluation showed no differences between the two groups. Since the methylation status can influence senescence, these two results agree [90]. Also, replicative senescence has been seen after the long-term culture of cells, which is different from the present study, where early cell passages were used [97]. Further experiments using higher passages will be important for determining whether RV-treated cells present with delayed senescence compared to GFs. Importantly, and fundamental for confirming the stem properties, the osteogenic, chondrogenic, and adipogenic differentiation capacities were evaluated, which are essential criteria for identifying MSCs [83]. The dedifferentiated cells were able to successfully differentiate into the three lineages, which supports not only their stem-like state, but also their interest for several regenerative fields. In future studies, evaluating the cell’s capacity to differentiate into other cell types would be of particular interest for expanding the possible applications of the dedifferentiated cells.

Finally, the safety of the dedifferentiated cells was determined using the teratoma formation model. While the dedifferentiated cells did not induce teratoma formation, the ESCs used as the positive control produced teratomas, as expected [98]. This is a fundamental result, since it allows for future cell transplantation protocols for tissue regeneration using dedifferentiated cells to be considered.

Taken together, these results represent one of the most comprehensive evaluations of dedifferentiated cells using RV as the dedifferentiation agent, and is the first report on the use of gingival fibroblasts as a cell source from which to obtain stem-like cells with uses in RD. The protocol used seems to have successfully induced a population with stem-like characteristics. Using an easily accessible, abundant, and healthy cell source, such as gingival tissue, is a major advantage. This method surpasses the limitations of conventional approaches to obtaining or inducing stem cells, providing patient-specific stem cell therapy without compatibility problems and eliminating technical, ethical, and security issues [32,42]. Furthermore, this molecular approach allows for the control of specific proteins involved in the dedifferentiation process without performing abnormal genetic modification, which is the basis of current iPSC generation methods. In this way, the cells’ malignant transformation risk is minimized compared to that of iPSCs [32]. Focusing on RD, GFs are obtained from the same micro-environment as other oral tissues, which may facilitate their differentiation towards them. Also, in addition to RD, the stem-like cells obtained could potentially be used for several other diseases where cell therapy is feasible. Despite these advantages, GFs present with important biological differences between individuals, and other factors, such as age, could compromise the results [99]. Collection from the oral cavity, in vitro manipulation, and the expansion of the cells also pose contamination risks, so the appropriate equipment, cultivation methods, and strict adherence to legislation are required to increase the patient’s safety. Also, considering the present study was developed with mouse cells, differences regarding human cells could exist. Therefore, the results should be confirmed using human cells to support their future clinical use.

Further studies should focus on improving the dedifferentiation efficiency by optimizing RV concentration, exposure time, or even chemical combinations. Additionally, isolating the cells that successfully dedifferentiate could allow for more pure stem-like cultures to be obtained, improving their further differentiation potential. This potential could be validated through the differentiation of other cell types, for instance, odontoblastic-like cells, which are fundamental for the regeneration of oral tissues.

## 5. Conclusions

The extensive characterization performed here shows that significant functional, morphological, and genetic changes occur during the dedifferentiation process. RV caused toxicity at higher concentrations, decreased cell metabolic activity and protein content, and induced genotoxicity and apoptosis. The cells obtained showed an enlarged size, polyploidy, and a cycle arrest in the G2/M phase. A subpopulation of GFs were successfully dedifferentiated into stem-like cells, as supported by the increased expression of CD90, CD 105, and TERT, the existence of a CD106+ population, and their trilineage differentiation capacity, which is of interest for RD procedures. The dedifferentiated cells did not induce teratoma formation.

## Data Availability

Data are contained within the article and the Supplementary Materials.

## Supplementary Materials

The following supporting information can be downloaded at: https://www.mdpi.com/article/10.3390/pharmaceutics16020207/s1, Figure S1: Cell cycle analysis; Figure S2: Channel images of alkaline phosphatase protein expression evaluated using immunocytochemistry; Figure S3: Teratoma formation by embryonic stem cells; Table S1: RV screening conditions.
