# Supplementary material for: In Vitro Characterization of Reversine-Treated Gingival Fibroblasts and Their Safety Evaluation after In Vivo Transplantation
Source: Pharmaceutics. 2024 Jan 31;16(2):207. doi: 10.3390/pharmaceutics16020207 (PMC10892828; doi:10.3390/pharmaceutics16020207)
Supplement: Supplementary file 1 [file pharmaceutics-16-00207-s001.zip › pharmaceutics-2810264-supplementary.pdf]

Table S1. Reversine screening conditions

Group	Reversine Concentration	Cell culture medium renewal (each 24h)	Number of reversine administrations
DMSO	0μM	No	0
1μM 1× WO	1μM	No	1
1μM 4× W	1μM	Yes	4
1μM 4× WO	1μM	No	4
2.5μM 1× WO	2.5μM	No	1
2.5μM 4× W	2.5μM	Yes	4
2.5μM 4× WO	2.5μM	No	4
5μM 1× WO	5μM	No	1
5μM 4× W	5μM	Yes	4
5μM 4× WO	5μM	No	4

1×: one administration. 4×: four administrations. W: with medium change; WO: without medium change

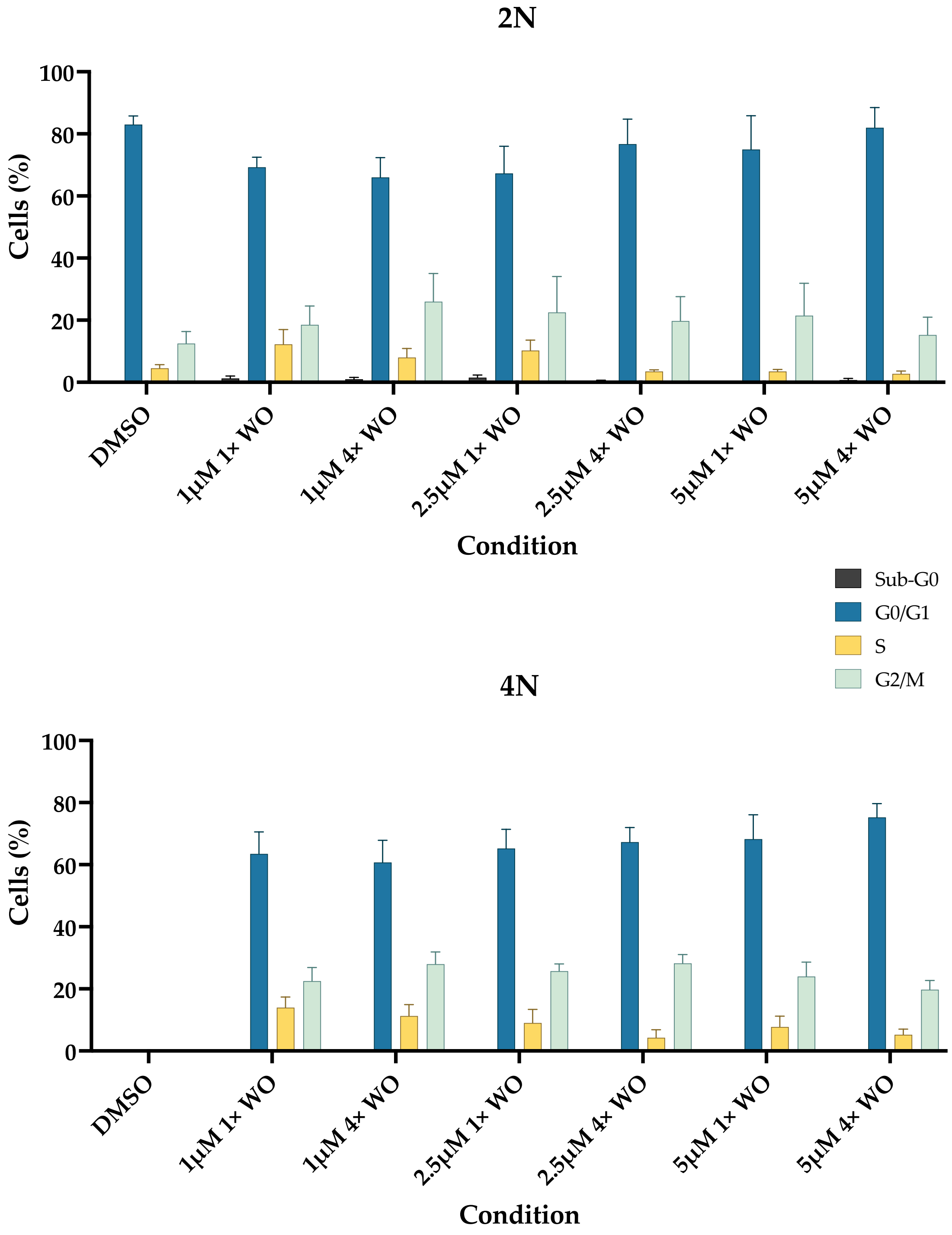

Figure S1: Cell cycle analysis. Percentage of cells in Sub-G0, G0/G1, S, and G2/M phases for the 2N and 4N populations. Results were obtained by flow cytometry and are presented as mean ± SE of at least three independent experiments. 1×: one administration. 4×: four administrations. WO: without medium change.

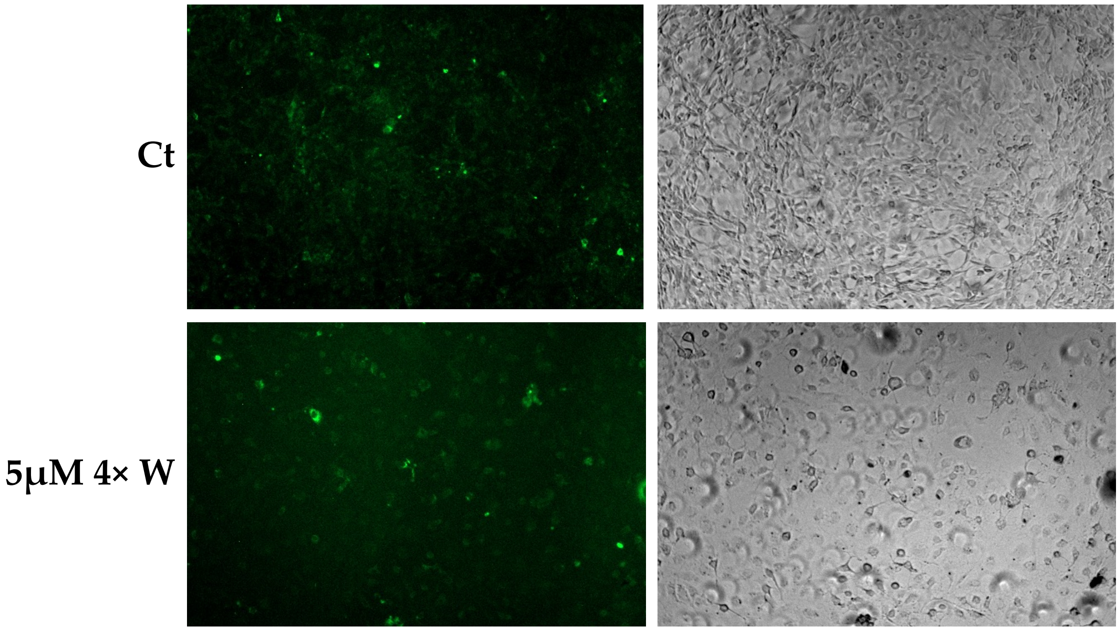

Figure S2. Channel images of the enzymatic activity labeling of alkaline phosphatase. The fluorescence (left column) and optical (right column) are shown for the control group and the dedifferentiated cells (5µM 4× W). Ct: control. 4×: four administrations. W: with medium change.

(a)

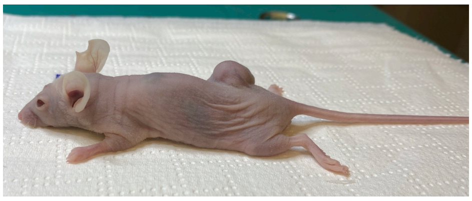

(b)

(c)

(d)

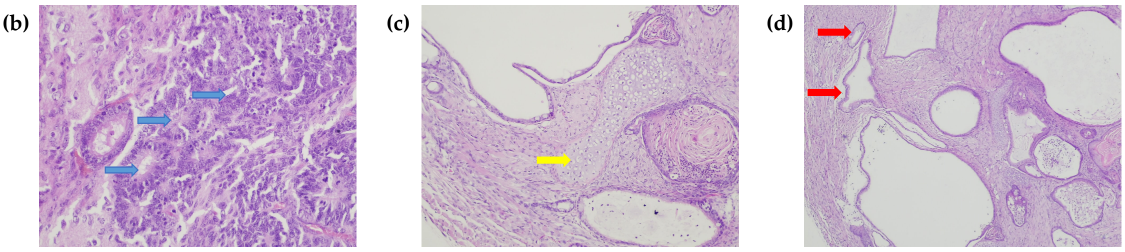

Figure S3. Teratoma formation **(a)** Teratoma formation occurred after subcutaneous injection of mouse embryonic stem cells (mESCs). Representative images of **(b)** mature and immature nervous tissue (ectoderm) – blue arrows, 200× magnification; **(c)** cartilage (mesoderm) – yellow arrows, 100× magnification and **(d)** glandular tissue (endoderm) – red arrows, 40× magnification are shown. Hematoxylin and eosin staining.
